# Phospho-Mimetic Mutation at Ser602 Inactivates Human TRPA1 Channel

**DOI:** 10.3390/ijms21217995

**Published:** 2020-10-27

**Authors:** Kristyna Barvikova, Ivan Barvik, Viktor Sinica, Lucie Zimova, Viktorie Vlachova

**Affiliations:** 1 Department of Cellular Neurophysiology, Institute of Physiology Czech Academy of Sciences, 142 20 Prague, Czech Republic; tynabarvikova@seznam.cz (K.B.); viktor.synytsya@fgu.cas.cz (V.S.); lucie.zimova@fgu.cas.cz (L.Z.); 2 Division of Biomolecular Physics, Institute of Physics, Faculty of Mathematics and Physics, Charles University, 121 16 Prague, Czech Republic; ibarvik@karlov.mff.cuni.cz

**Keywords:** transient receptor potential ankyrin 1, TRP channel, protein kinases, phosphomimetic, mutagenesis, phosphorylation

## Abstract

The Transient Receptor Potential Ankyrin 1 (TRPA1) channel is an integrative molecular sensor for detecting environmental irritant compounds, endogenous proalgesic and inflammatory agents, pressure, and temperature. Different post-translational modifications participate in the discrimination of the essential functions of TRPA1 in its physiological environment, but the underlying structural bases are poorly understood. Here, we explored the role of the cytosolic N-terminal residue Ser602 located near a functionally important allosteric coupling domain as a potential target of phosphorylation. The phosphomimetic mutation S602D completely abrogated channel activation, whereas the phosphonull mutations S602G and S602N produced a fully functional channel. Using mutagenesis, electrophysiology, and molecular simulations, we investigated the possible structural impact of a modification (mutation or phosphorylation) of Ser602 and found that this residue represents an important regulatory site through which the intracellular signaling cascades may act to reversibly restrict or “dampen” the conformational space of the TRPA1 channel and promote its transitions to the closed state.

## 1. Introduction

Phosphorylation is supposed to be one of the most important post-translational mechanisms reversibly regulating the Transient Receptor Potential Ankyrin 1 (TRPA1) channel under various physiological and pathophysiological conditions [1,2]. This channel is abundantly expressed in a subset of nociceptive somatosensory neurons where it acts as a polymodal detector of diverse pain-producing chemical and physical stimuli [3,4]. The expression of TRPA1 is also detected in a wide variety of non-neuronal cells [5,6,7,8], such as lung fibroblast and epithelial cells, smooth muscle cells [9,10], hair cells [11], astrocytes [12], oligodendrocytes [13], Schwann cells [14], odontoblasts [15], and synoviocytes [16], indicating the involvement of the channel in the (patho)physiology of multiple organ systems. Activation of TRPA1 produces an acute nociceptive response through peripheral release of neuropeptides from sensory nerve endings or from non-neural tissues. Under a number of chronic conditions associated with inflammation, tissue damage, or oxidative stress, endogenous substances released at the site of injury modulate TRPA1 via the G-protein-coupled receptor and phospholipase C-coupled signaling cascades. Among such agents are extracellular proteases, prostaglandins, bradykinin, serotonin, substance P, calcitonin gene related peptide, nerve growth factor (NGF), tumor necrosis factor α (TNFα), and many more [17,18,19,20]. These mediators can directly activate TRPA1 [21,22,23] or stimulate protein kinase A (PKA) [24,25,26,27], protein kinase C (PKC) [25], p38 mitogen-activated protein kinases [28], cyclin dependent kinase 5 [29,30], or phospholipase C (PLC) pathways [24,31] to induce phosphorylation of TRPA1. Phosphorylation, in turn, significantly alters the activation threshold and/or membrane expression of TRPA1, amplifying the inflammation and rendering nociceptors more sensitive to noxious stimulation.

For effective phosphorylation by PKA and PKC, TRPA1 needs the presence of the scaffolding A-kinase anchoring protein 79/150 (AKAP) [25]. AKAP directly interacts with TRPA1 [32], spatially constrains phosphorylation, and most likely serves as a molecular hub that contributes to the efficiency and specificity of the cellular signaling network regulating the channel under various physiological or pathophysiological conditions [33]. Importantly, the sensitization of TRPA1 through the PKA-AKAP pathway is responsible for persistent mechanical hypersensitivity [25], carboplatin-induced mechanical allodynia, and cold hyperalgesia [34]. While most of the kinases described so far that act on TRPA1 functionally up-regulate the channel’s function, 5’ adenosine monophosphate-activated protein kinase (AMPK), on the contrary, down-regulates TRPA1 plasma membrane expression in dorsal root ganglion neurons which might represent a potential mechanism underlying painful diabetic peripheral neuropathy and other diseases with a similar pathophysiological profile of metabolic dysfunction [35]. In addition, several tyrosine residues located at the N-terminus have been proposed to be involved in the inhibition of TRPA1 by Src kinase in SH-SY5Y human neuroblastoma cells [36]. Despite the presumed physiological importance of the molecular mechanisms that establish and control the subtle balance between phosphorylated and dephosphorylated states of TRPA1, information regarding the specific sites underlying these processes remains scarce (reviewed in [1,18,37,38]).

**Figure 1:**
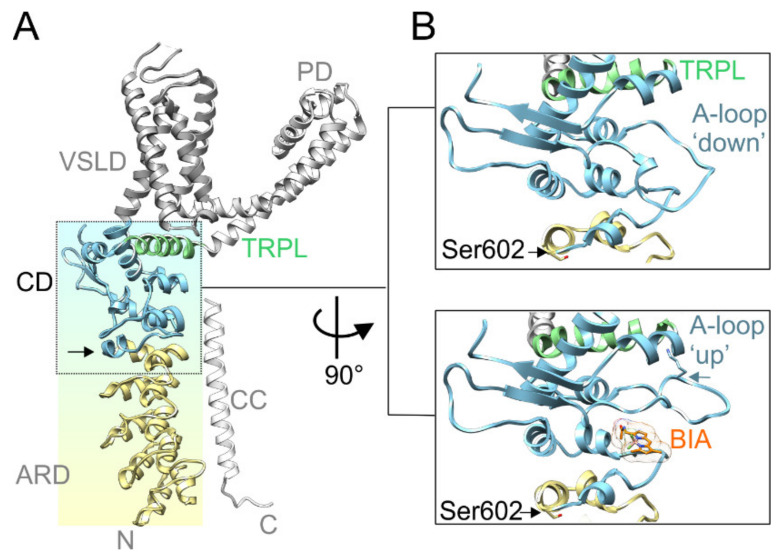
Serine 602 (Ser602) precedes the functionally key coupling domain. (**A**) The structure overview of a Transient Receptor Potential Ankyrin 1 (TRPA1) subunit (PDB ID: 6V9W [39]) with indicated voltage-sensor like domain (VSLD; grey ribbon), pore domain (PD; light grey ribbon), coupling domain, also dubbed as allosteric nexus (CD; light blue ribbon), ankyrin repeat domain (ARD; yellow ribbon), C-terminal coiled-coil domain (CC; white ribbon), and TRP-like domain (TRPL; light green ribbon). Black vertical arrow indicates the position of Ser602. (**B**) Close-up view of the coupling domain in two conformations: Upper box, activation loop (A-loop) in a “down” conformation (PDB ID: 6V9W). Lower box, activation loop in an ”up” conformation (PDB ID: 6V9V), enabling binding of BODIPY-iodoacetamide (BIA) and stabilization of the A-loop through the interaction of Lys671 (indicated by horizontal blue arrow) with backbone carbonyls at the C-terminus of the TRP-like helix. Black horizontal arrows indicate position of Ser602.

Recently, it has been proposed that natural or exogenous thiol-reactive electrophilic compounds activate TRPA1 through a two-step mechanism that involves conformational changes within a specific cytoplasmic membrane proximal domain (Figure 1A). This functionally key part of the protein, dubbed the allosteric nexus [39] or coupling domain [40], is composed of seven N-terminal and one C-terminal short helices, three β-strands that form an antiparallel β-sheet, and the TRP-like helix. In the process of electrophile-induced activation, covalent modification of Cys621 is followed by partial modification of Cys665 and repositioning of Lys671 that is stabilized through its interaction with backbone carbonyl oxygens at the C-terminus of the TRP-like domain, leading to pore widening [39]. Structurally, the allosteric nexus is immediately preceded by Ser602 (Figure 1B), a residue from ankyrin repeat 16 that is predicted by various servers [41] to be a potential phosphorylation site for several kinases, including AGC, CMGC, and Ca^2+^/calmodulin-dependent protein kinase families. Interestingly, the side chain of this serine is exposed to the cytoplasm in TRPA1 structures obtained in the ligand-free state (PDB ID: 6PQQ; [40]) or in the presence of the covalent agonists allyl isothiocyanate (3J9P; [42]), benzyl isothiocyanate (6PQP; [40]), and JT010 (6PQO; [40]). In contrast, in other structures resolved recently in various closed and activated conformations (6V9V, 6V9W, 6V9X, and 6V9Y; [39]), the side chain of Ser602 is oriented towards the protein by forming hydrogen bonds with main chain carbonyls. This indicates that the role of this putative consensus phosphorylation site may depend on the conformational state of the channel. We reasoned that being located near the functionally important allosteric nexus, a modification of Ser602 may have a strong impact on TRPA1 channel gating. Therefore, in this study, we explored the role of this residue as a potential target of phosphorylation.

## 2. Results

### 2.1. Phophomimetic Mutation S602D Abrogates Voltage-Dependent Activation of TRPA1

**Figure 2:**
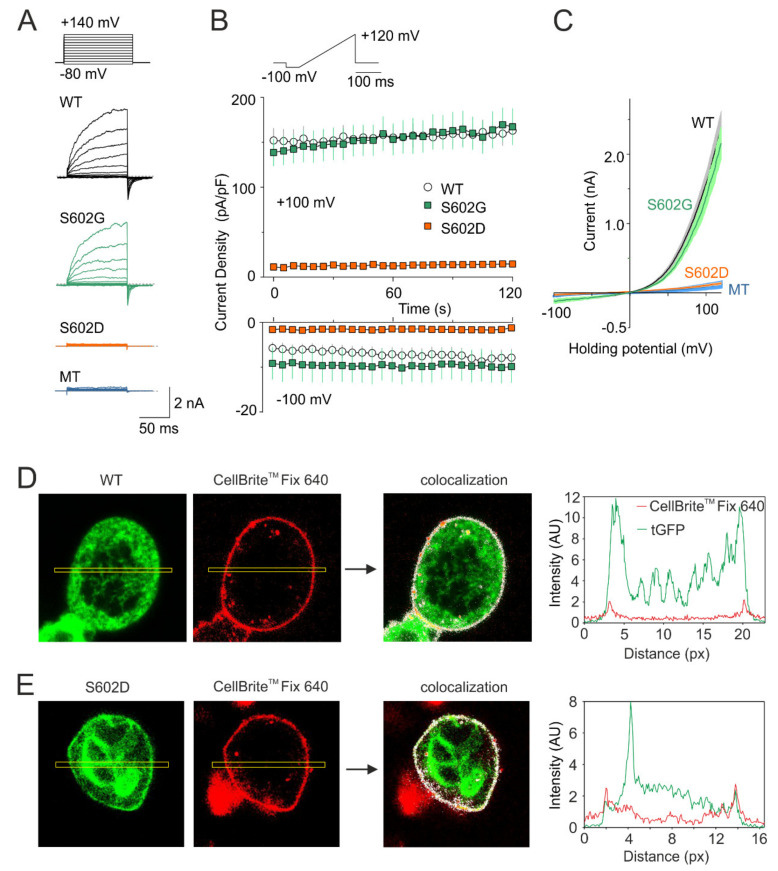
Mutation S602D abrogates voltage-dependent activation of TRPA1. (**A**) Currents through wild-type TRPA1 (WT), S602G, and S602D channels and currents from HEK 293T cells transfected with control plasmid (MT) induced by voltage-step protocol (shown above): 100-ms steps from −80 mV to +140 mV (increment +20 mV), holding potential −70 mV. (**B**) Average whole-cell current densities induced by depolarizing voltage measured from HEK 293T cells expressing TRPA1 (white circles indicating mean ± S.E.M.; *n* = 21), S602G (green squares ± S.E.M.; *n* = 10), or S602D (orange squares ± S.E.M.; *n* = 9). Voltage ramp protocol (shown in upper trace) was applied repeatedly each 5 s for 3 min. Amplitudes were measured at −100 mV and +100 mV and plotted as a function of time. (**C**) Mean current-voltage relations (mean as thin darker curves, ± SEM as lighter-colored envelopes) plotted for the initial response from cells expressing wild-type TRPA1, S602G, S602D or mock-transfected (MT) cells, exposed to the protocol shown in B. (**D**,**E**) Fluorescence colocalization analysis of HEK 293T cells transfected with tGFP-tagged (green) wild-type TRPA1 (D) or S602D mutant (E). Images are shown at 130× magnification. Cells were stained with the membrane dye CellBrite^TM^ Fix 640 (red). An ImageJ plugin Colocalization Finder (see the Methods section) was used to highlight corresponding pixels on a RGB overlap (colocalization image indicated by arrow). Right graphs: the fluorescence intensity profiles (in arbitrary units, AU) were measured along rectangle drawn across the cell shown left. Similar results were obtained from nine WT-expressing and five S602D-expressing cells from three independent transfections.

In order to explore the role of Ser602 as a potential phosphorylation site, we constructed the phosphomimetic and phoshonull mutants of human TRPA1, S602D, and S602G, and tested their voltage-dependent activation properties in transiently transfected HEK 293T cells under whole-cell patch clamp conditions (Figure 2). At the beginning of the recording from each cell, we ensured that a series of depolarizing pulses from −80 mV to +140 mV applied in the presence of extracellular control solution produced typical outwardly rectifying membrane currents (Figure 2A). Afterwards, we repeatedly applied voltage ramps from −100 mV to +120 mV (1 V/s; holding potential −70 mV) with 5-s intervals (Figure 2B,C). Whereas S602G exhibited outwardly rectifying membrane currents that were not significantly different from wild-type TRPA1 (*n* = 10), the S602D construct was nonfunctional in all cells tested within a recording period of at least 120 s (*n* = 9). To investigate if the S602D mutation affects the surface expression of TRPA1, we expressed tGFP-tagged wild-type TRPA1 or S602D in HEK 293T cells and performed confocal microscopy by using CellBrite^TM^ Fix 640 to visualize plasma membrane (Figure 2D,E). The colocalization analysis of fluorescence intensity profiles measured along a rectangle drawn across the cell membrane confirmed that the areas of high intensity were observed at the cell periphery similarly in both constructs. Although not quantitatively, together these findings indicate that the S602D mutant is significantly expressed on the cell membrane, but it is not active.

### 2.2. Salt-Bridge Formation is Not Involved in the Effects Caused by the S602D Mutation

**Figure 3:**
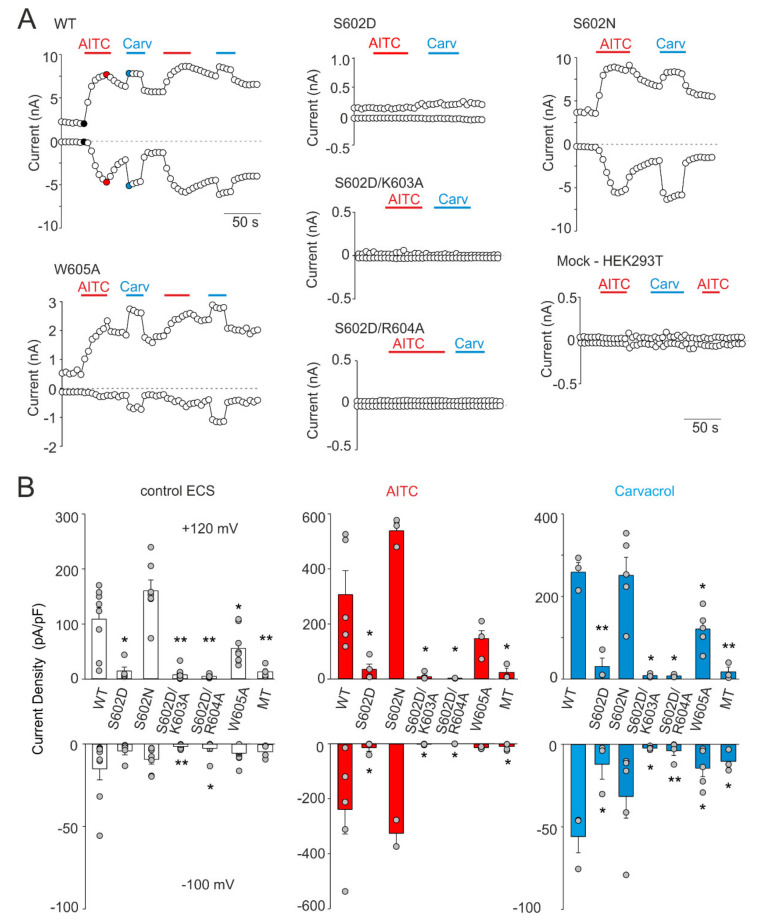
Effects of mutations at Ser602 and contiguous residues on TRPA1 responses. (**A**) Time course of whole-cell current responses induced by voltage ramp (−100 mV, +120 mV; protocol shown in Figure 2B), applied in the presence of extracellular control solution (ECS), allyl isothiocyanate (AITC; 100 µM) or carvacrol (Carv; 50 µM). (**B**) Maximal amplitude of currents measured from recordings such as shown in A for indicated mutants (*n* = 3–10) and for mock-transfected cells (MT). The amplitudes were measured at times indicated by the colored points shown for the wild-type (WT) in A. The error bars represent the standard error of the mean. * *p* < 0.05; ** *p* < 0.005; Student’s *t*-test.

The structural role of Ser602 in TRPA1 channel functioning was next investigated by substituting this residue with asparagine, a polar amino acid of a similar size as negatively charged aspartate. We also evaluated the possibility that the disruptive effect of S602D may be caused by salt-bridge formation with proximal residues Lys603 or Arg604. Moreover, we tested the structural role of Trp605, a residue contained in the allosteric nexus. According to various prediction servers (e.g., http://cam.umassmed.edu/ and http://calcium.uhnres.utoronto.ca/), the region around Trp605 may represent not only a putative phosphorylation site but also a putative consensus binding site for Ca^2+^/calmodulin [33]. Figure 3 summarizes the whole-cell current measurements from the S602D, S602N, S602D/K603A, S602D/R604A, and W605A mutants. To test the general role of these residues in channel gating, we examined not only the sensitivity of the constructs to depolarizing voltage and the electrophilic agonist allyl isothiocyanate (AITC; 100 µM), but also to the nonelectrophilic agonist carvacrol (50 µM) that activates the channel through a different, more classical noncovalent mechanism. The S602D mutant failed to produce any appreciable currents in response to agonist stimulation and its function was not rescued by either the K603A or R604A mutation. The S602D mediated currents measured in extracellular control solution or in the presence of AITC or carvacrol were not significantly different from the currents measured in mock-transfected cells (*p* = 0.905, 0.682 and 0.620 at +120 mV and *p* = 0.970, 0.676 and 0.845 at −100 mV; *n* = 4). The currents through S602N were not significantly different from wild-type channels (Figure 3B). The W605A mutant was sensitive to both agonists, although its responses to carvacrol were significantly lower than in wild-type channels (*p* = 0.006; *n* = 5), which indicates structural involvement of the tryptophan 605 in the process of channel activation. These data together suggest that the introduction of a negatively charged amino acid at position 602 mimics the phosphorylated state of the channel and that possible phosphorylation at Ser602 may cause the channel to be irresponsive to voltage and chemical stimuli.

### 2.3. Molecular Dynamics Simulations of the Phosphorylation of TRPA1 at Ser602

**Figure 4:**
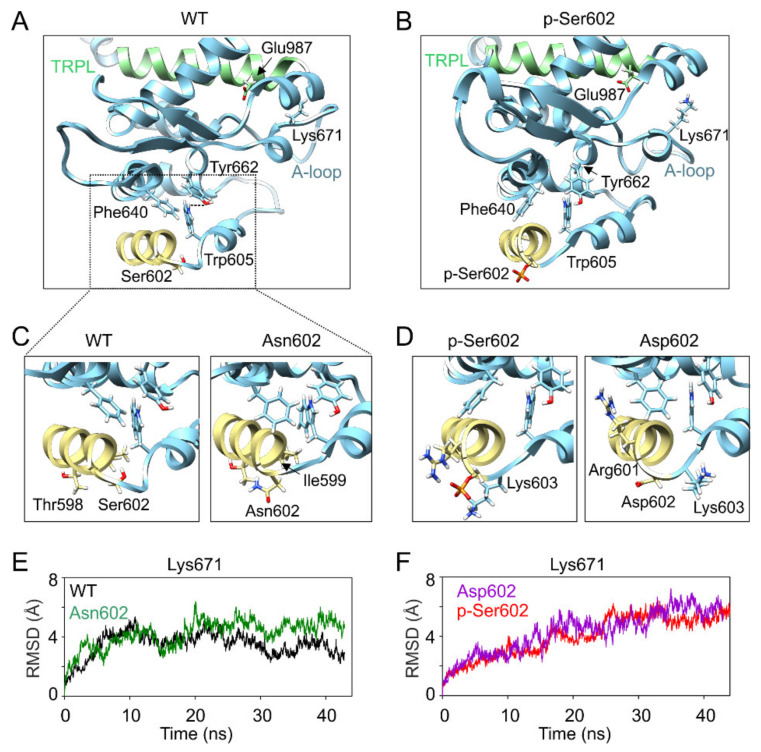
MD simulations indicate possible structural changes induced by modification at Ser602. (**A**,**B**) Close up view of the cluster of bulky amino acids Phe640, Trp605, and Tyr662 (chain B) through which the signal from Ser602 can be transduced to the activation loop (A-loop). Distances between Trp605 and Tyr662 shown in Supplementary Figure S2 were measured as indicated by dashed line in A. (**C**,**D**) Close up view of the local conformations of the outer helix of ankyrin repeat 16 obtained from MD simulations of the indicated constructs. (**E**,**F**) Time course of the average root mean square deviation (RMSD) values for Lys671, measured from the indicated TRPA1 constructs.

To further investigate how conformational changes induced by modification of Ser602 can be transduced to the channel gate, we conducted molecular dynamics (MD) simulations using the published cryo-electron microscopic structure of human TRPA1 captured in the open, ligand-occupied state (PDB ID: 6V9X; [39]). The simulations were performed with the “wild-type” structure, the S602D and S602N mutants, and with the phosphorylated state of Ser602, p-Ser602 (Figure 4, Supplementary Figures S1–S3).

The molecular dynamics (MD) trajectory reached 42–44-ns and the root mean square deviation (RMSD) measured for individual subunits and for the channel complex indicated that the models were stable (Supplementary Figure S1). Obvious differences were observed in local conformations around the position 602 among the wild-type, S602N, S602D, and the p-Ser602 structures (Figure 4A–D). In the wild-type structure and in S602N, the side chain of the amino acid at position 602 forms H-bonds with backbone carbonyl groups of Thr598 and Ile599 from the outer helix of ankyrin repeat 16 (AR16) (Figure 4A–C). In contrast, p-Ser602 phosphorylation and the S602D mutation orient the side chain into the aqueous environment where it forms salt bridges with the neighboring Lys603 (Figure 4B,D). In S602D, the backbone NH group of Asp602 interacts with the backbone carbonyl groups of Thr598 and Ile599 which slightly prolongs the end of the outer helix of AR16. Notably, this is similar to the interactions observed in the TRPA1 structure 3J9P [42], which has been obtained in the presence of covalent agonist but captured the channel in a closed state. In p-Ser602, the phosphoryl group forms extensive contacts with Arg601, a residue that interacts with Phe640 and through which the signal can be transmitted upwards through the allosteric nexus. On the other hand, such an extensive contact network was not observed in the S602D structure, suggesting that Arg601 is not the key to explaining the main impact of the mutation and/or phosphorylation.

Figure 4A shows that Phe640 forms a cluster of bulky amino acids with neighboring Trp605 and Tyr662. The relative positions of these two amino acids usually did not change much over the wild-type and S602N MD simulations (Supplementary Figure S2) so that their distances were less than 3 Å for about 50% and 42% of MD simulation time in three subunits. Conversely, in the case of S602D and p-Ser602, the changes in the distances were more pronounced (7.5% and 22% respectively). These changes appear to be efficiently transmitted to the crucial TRP-like helix, so they can undoubtedly influence channel gating. Specifically, this can be seen in the time evolution of the average root mean square deviation of Lys671 (Figure 4E,F) and the time course of the distances between amino acids Lys671 and Glu987 (measured between Cα atoms, Supplementary Figure S3). While in the case of wild-type and S602 structures, these distances were below ~10 Å for about 60% of the MD simulation time in all subunits, in the case of S602D and p-Ser602, the relative time spent below ~10 Å was only 26% and 30%, respectively. These results suggest that the possible structural mechanism through which the modification at Ser602 produces inactivation of the channel involves changes in the coordination between Ly671 and the TRP-like helix, which are the critical components of the channel gating machinery.

## 3. Discussion

Our present data show that the single mutation of Ser602 to aspartate renders the TRPA1 channel inactive, whereas the replacement of this serine with asparagine, a residue of a similar side-chain as aspartate but lacking its negative charge, or with a flexible glycine, fully retains channel functionality. The data show further that the phosphomimetic mutant S602D is expressed on the plasma membrane, and thus appears to retain key protein functions. These results raise the possibility that Ser602 represents a potential phosphorylation site through which specific intracellular signaling cascade(s) may act to promote the transition of the channel to the closed state.

Although phosphomimetic/phosphonull substitution studies have proved to be instrumental and highly informative in obtaining new insights into the mechanisms of TRPA1 regulation by phosphorylation [26,29,36,43,44], the results should be interpreted with caution. First, the negative charge introduced by aspartate substitution (−1) does not match that of the phosphorylated residue (generally, −1.5) at physiological pH. Second, if the Ser602 serves as a recognition signal for an adaptor protein, a phosphomimetic mutant should not fit into its binding pocket. The third significant aspect is that a phosphomimetic substitution mimics long-term protein phosphorylation. Physiologically, phosphorylation is a dynamic process, often transient in response to various stimuli or cellular signaling events [45,46]. Thus, due to steric and intrinsic activity differences, the aspartate mutation does not necessarily imitate the effects of a phosphorylated residue. Our molecular simulations indicate that the structural impact of covalently attached phosphate produced by a phosphate group at position 602 is very similar to that of negatively charged amino acid aspartate, and so the overall chemical environment introduced by the mutation can be similar to phosphorylation. The proximity of Ser602 to the functionally crucial allosteric nexus makes it tempting to speculate that conformational changes at or near this residue may readily propagate to the gates of the channel. Indeed, our molecular simulations are in agreement with our functional data and suggest that the negative charge introduced by aspartate substitution at position 602 significantly alters local conformation of the activation loop through affecting the interaction of Trp605, Phe640, and Tyr662 and, subsequently, weakening the coordination between Lys671 and backbone carbonyl oxygens at the TRP-like domain C-terminus. Our electrophysiological data demonstrate that substitution of Trp605 by alanine notably reduced but did not abolish voltage- and carvacrol-induced activation (Figure 3). This suggests that Trp605 is important but not necessary for channel activation. Structural comparisons indicate that Tyr662 is located in a completely different position in the 3J9P structure of the closed TRPA1 [42]. In fact, Tyr662 is shifted several tens of Ångströms from the place where we observe it in the 6V9X structure. It is therefore possible that the conformational changes observed by us—with respect to the relative position of Trp605 and Tyr662 in the S602D and p-Ser602 MD simulations (see Supplementary Figure S2)—could ultimately lead to a broader conformational transition that could promote channel closure.

More than 900,000 different protein phosphorylation sites, including predicted, have been reported in various literature-curated databases and numerous bioinformatics resources and tools are available for the prediction of phosphorylation sites (see [41] and references therein). However, despite intensive research over many years, only about a few percent (~3%) of phosphorylation sites reported have been attributed to an experimentally confirmed human kinase and have their biological role clearly identified [41,47,48]. At least, the present state of knowledge enables to identify several kinases that may phosphorylate TRPA1 at Ser602 using various phosphorylation site prediction tools (reviewed in [41]; Table 1).

Most likely, the protein kinases A and C can be excluded from consideration because direct activation of protein kinase A (by 8-Br-cAMP or forskolin) or protein kinase C (by phorbol 12-myristate 13-acetate or phorbol 12,13-dibutyrate) has been already shown to up-regulate TRPA1 [24,25,26], which is opposite to the observed effects of the S602D mutation. Recent technological advances in mass spectrometry-based phosphoproteomic profiling are steadily increasing the scope of a global exploration of protein phosphorylation [46,48]. A key focus for future research can be to experimentally assess the phosphorylation status of Ser602 under various physiologically relevant conditions.

The enormous variability of the mechanisms found in relation to the role of TRPA1 in various physiological processes is a major challenge for the future. Most of the kinases described so far act on TRPA1 to functionally up-regulate the channel’s function. The region around Ser602, on the contrary, might represent a potential site from which the activity of the channel can be effectively down-regulated under some pathophysiological conditions. Future studies, perhaps using a co-expression of TRPA1 with the predicted kinases (see Table 1) or examining various kinase modulators and mass spectrometry in a native system (i.e., DRG neurons), will be required to determine whether the mechanisms regulating TRPA1 channel involve Ser602. One of the general aspects that are usually considered when deciding whether or not the identified residue may represent a potential phosphorylation site is its evolutionary conservation [55,56]. Ser602 is not conserved across all species but is completely conserved in primates including human and any missense polymorphism at this site and the neighboring Lys603 have been identified in human. On the other hand, a total of 14 missense variants in the *TRPA1* gene have been detected at positions immediately preceding and following these two residues: T598M (rs147715599), I599V (rs145600263), I599N (rs745749488), I600V (rs1236229851), R601S (rs148585412), R601T (rs1347666288), R604I (rs377764138), R604T, W605R (rs748788882), D606N, D606Y (rs1352827754), E607K (rs755681762), C608Y (rs747708313), and C608R (rs955642376). In particular, some of these polymorphisms may affect the phosphorylation consensus or gating and deserve further investigation to elucidate their potential involvement in human pathophysiology.

## 4. Materials and Methods

### 4.1. Cell Culture, Constructs, and Transfection

Human embryonic kidney 293T cells (HEK 293T; ATCC, Manassas, VA, USA) were cultured in Opti-MEM I media (Invitrogen, Carlsbad, CA, USA) supplemented with 5% fetal bovine serum. The magnet-assisted transfection (IBA GmbH, Gottingen, Germany) technique was used to transiently co-transfect the cells in a 15.6 mm well on a 24-well plate coated with poly-l-lysine and collagen (Sigma-Aldrich, Prague, Czech Republic) with 200 ng of green fluorescent protein (GFP) plasmid (TaKaRa, Shiga, Japan) and with 400 ng of cDNA plasmid-encoding wild-type or mutant human TRPA1 (pCMV6-XL4 vector, OriGene Technologies, Rockville, MD, USA) or control plasmid. The cells were used 24–48 h after transfection. At least three independent transfections were used for each experimental group. The wild-type channel was regularly tested in the same batch as the mutants. The mutants were generated by PCR by using a QuikChange II XL Site-Directed Mutagenesis Kit (Agilent Technologies, Santa Clara, CA, USA), and they were confirmed by DNA sequencing (Eurofins Genomics, Ebersberg, Germany).

### 4.2. Electrophysiology

Whole-cell membrane currents were filtered at 2 kHz by using the low-pass Bessel filter of the Axopatch 200B amplifier, and they were digitized (5–10 kHz) with a Digidata 1440 unit and the pCLAMP 10 software (Molecular Devices, San Jose, CA, USA). Patch electrodes were pulled from borosilicate glass and heat-polished to a final resistance of between 3 and 5 MΩ. Series resistance was compensated by at least 60%. The experiments were performed at room temperature (23–25 °C). Only one recording was performed on any one coverslip of cells to ensure that recordings were made from cells that had not been previously exposed to chemical stimuli. Extracellular bath solution was Ca^2+^-free and contained: 140 mM NaCl, 5 mM KCl, 2 mM MgCl_2_, 5 mM EGTA (ethylene glycol-bis (β-aminoethyl ether)-N,N,N’,N’-tetraacetic acid), 10 mM 4-(2-Hydroxyethyl)piperazine-1-ethanesulfonic acid (HEPES), 10 mM glucose, and pH 7.4 was adjusted by tetramethylammonium hydroxide. The intracellular solution contained 125 mM Cs-gluconate, 15 mM CsCl, 5 mM EGTA, 0.5 mM CaCl_2_, 10 mM HEPES, 2 mM adenosine 5′-triphosphate magnesium salt, 0.3 mM guanosine 5′-triphosphate sodium salt hydrate, adjusted with CsOH to pH 7.4. The chemicals were purchased from Sigma-Aldrich (Prague, Czech Republic). The current-voltage relationships were recorded by using 50-ms steps ranging from holding potential of –70 mV to –100 mV, followed by a 220-ms ramp from –100 mV to +120 mV.

### 4.3. Confocal Microscopy

HEK 293T cells transfected with wild-type or S602D mutant of human TRPA1 tagged with tGFP (turbo GFP; Origene Technologies, Rockville, MD, USA) were stained with the cytoplasmic membrane labeling dye CellBrite^TM^ Fix 640 (Biotium, Fremont, CA, USA) according to the manufacturer’s protocol. Briefly, the cells were incubated in phosphate-buffered saline (1 mL) containing the dye (1 µL) for 20 min at 37 °C in a 5% CO_2_. After treatment, cells were washed and imaged in Opti-MEM I media (Invitrogen, Carlsbad, CA, USA) supplemented with 5% fetal bovine serum. The colocalization analysis was performed using the Colocalization Finder plugin in ImageJ and fluorescence intensity profiles measured along a rectangle drawn across the cell membrane.

### 4.4. Molecular Modeling

To elucidate the possible structural mechanism through which the substitution S602D produces inactivation of the channel and compare the impact of phosphorylation at Ser602, we used the human TRPA1 structure with PDB ID: 6V9X, determined by cryo-electron microscopy [39]. First, the mutations S602D and S602N were created using the „Mutate residue“ plugin in VMD [57]. The phosphorylated state of Ser602 (dianionic phosphate group) was created using the “Automatic PSF Builder” plugin with an appropriate parameter file (toppar_all36_prot_na_combined.par) within VMD software. The TRPA1 tetrameric structures (wild-type, mutated and phosphorylated) were inserted into the patch of the 1-palmitoyl-2-oleoylphosphatidylcholine (POPC) bilayer and solvated in transferable intermolecular potential 3-point (TIP3P) [58] water molecules to ensure at least 10 Å of solvent on both sides of the membrane and ionized in 0.5 M NaCl. This gives a periodic box size of 120 × 120 × 160 Å for a simulated system consisting of ~225,000 atoms. All atom structure and topology files were generated using VMD software [57]. Forces were computed using CHARMM27 force field for proteins, lipids, and ions [59,60,61]. All molecular dynamics (MD) simulations were produced with the aid of the software package NAMD2.13 [62] running on computers equipped with NVIDIA graphics processing units. First, Langevin dynamics was used for temperature control with the target temperature set to 310 K; the Langevin piston method was applied to reach an efficient pressure control with a target pressure of 1 atm [62]. The integration timestep was set to 2 fs. Simulated systems were energy-minimized, heated to 310 K, and production MD runs reached a length of 42–44 ns. Data were recorded every 20 ps and distances and contacts were analyzed using CPPTRAJ module from Amber Tools suite [63]. MD trajectories were visualized with the aid of the VMD 1.9 software package [57]. Figures were produced with the software packages UCSF Chimera 1.13 [64] and CorelDraw X7 (Corel Corporation, Otawa, Canada).

## 5. Conclusions

Our data identify an important N-terminal serine residue from which the channel functioning can be abrogated by a phospho-mimetic mutation. Beyond that, our data suggest that conformational changes produced by a substitution of Ser602 affect not only electrophilic- but also non-electrophilic- and voltage-dependent activation. This indicates that these activation mechanisms may converge and involve changes in the coordination between Ly671 and the TRP-like helix. Whether this serine residue indeed represents a phosphorylation site in vitro and in vivo is an interesting topic for future studies.

## Figures and Tables

**Table 1:** Protein kinases predicted at a high or medium stringency level/threshold to phosphorylate human Transient Receptor Potential Ankyrin 1 (TRPA1) at Ser602.

Predicted Protein Kinase	Prediction Server
Protein kinase A	GPS 5.0 [49], PhosphoPICK [50]
Protein kinase C	GPS 5.0, NetPhos [51], NetPhorest, NetworKIN [52]
Protein kinase D1	GPS 5.0 [49], PhosphoPICK [50]
Glycogen synthase kinase-3	GPS 5.0 [49]
AKT2 kinase	GPS 5.0 [49]
Rho-associated protein kinase	PhoScan [53]
Mitogen-activated protein (MAP) kinase 3	Musite [54]
MAP kinase-activated protein kinase 5	GPS 5.0 [49]
ULK1 protein kinase	GPS 5.0 [49]

## Supplementary Materials

Supplementary Materials can be found at https://www.mdpi.com/1422-0067/21/21/7995/s1.

## Abbreviations

TRPA1	Transient receptor potential ankyrin subtype 1
JT010	2-chloro-N-(4-(4-methoxyphenyl)thiazol-2-yl)-N-(3-methoxypropyl)-acetamide
AGC	Kinase family named after the protein kinase A, G, and C families
CMGC	Kinase family named after the initials of its subfamily members, including cyclin-dependent kinase, mitogen-activated protein kinase, glycogen synthase kinase and CDC-like kinase
AR16	Ankyrin repeat 16
