# Supplementary material for: Phospho-Mimetic Mutation at Ser602 Inactivates Human TRPA1 Channel
Source: Int J Mol Sci. 2020 Oct 27;21(21):7995. doi: 10.3390/ijms21217995 (PMC7663402; doi:10.3390/ijms21217995)
Supplement: Supplementary file 1 [file ijms-21-07995-s001.pdf]

Supplementary Materials

Phospho-Mimetic Mutation at Ser602 Inactivates Human TRPA1 Channel

Kristyna Barvikova [1], Ivan Barvik [2], Viktor Sinica [1], Lucie Zimova [1] and Viktorie Vlachova [1,*]

[1] Department of Cellular Neurophysiology, Institute of Physiology Czech Academy of Sciences, 142 20 Prague, Czech Republic; tynabarvikova@seznam.cz (K.B.); viktor.synytsya@fgu.cas.cz (V.S.); lucie.zimova@fgu.cas.cz (L.Z.)

[2] Division of Biomolecular Physics, Institute of Physics, Faculty of Mathematics and Physics, Charles University, 121 16 Prague, Czech Republic; ibarvik@karlov.mff.cuni.cz

* Correspondence: viktorie.vlachova@fgu.cas.cz; Tel.: +420-29644-2711

Received: 29 September 2020; Accepted: 26 October 2020; Published: 27 October 2020

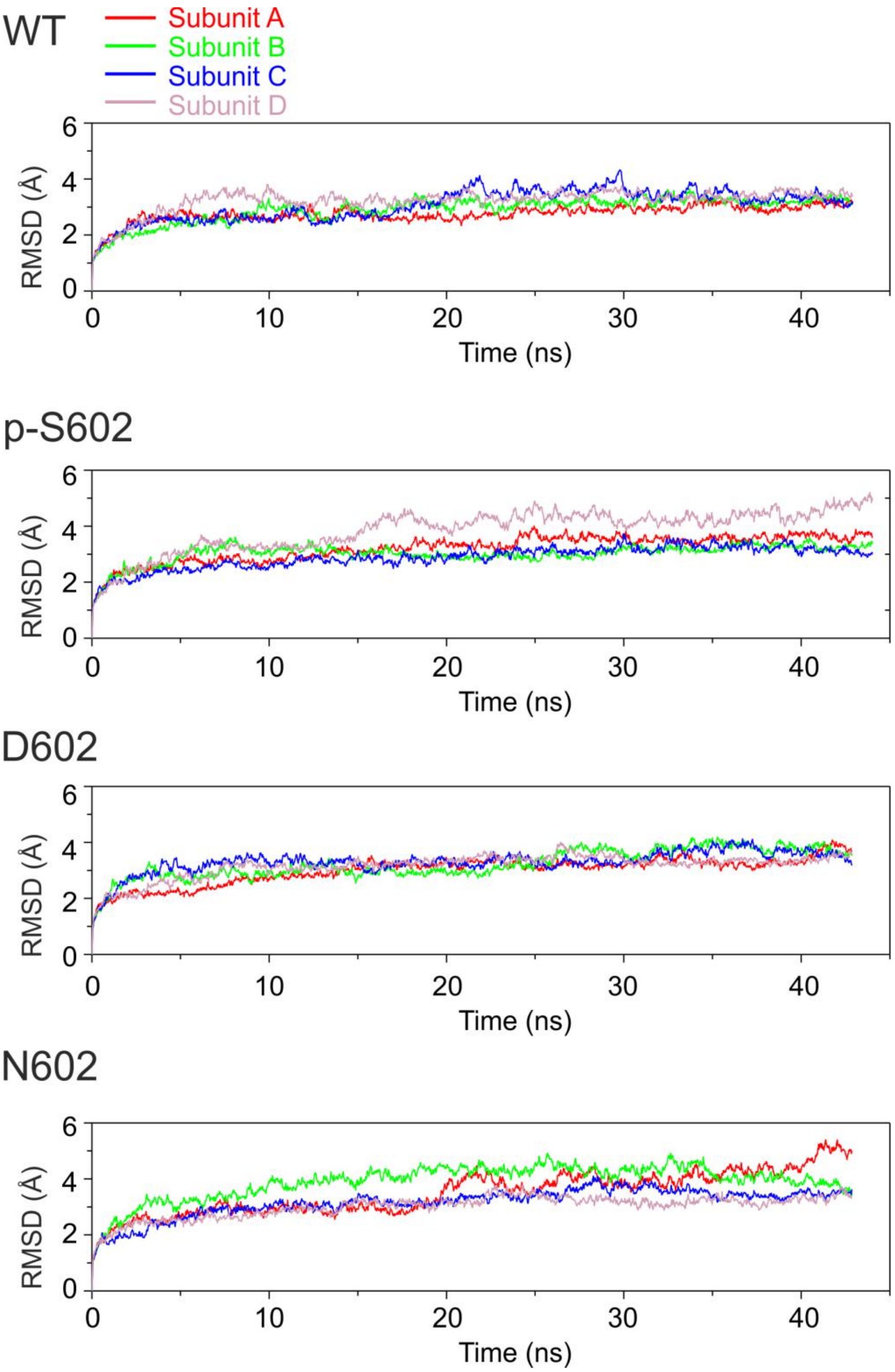

Figure S1. Molecular dynamics simulation results obtained for TRPA1 models based on 6V9X structure. Time evolution of the root mean square deviation (RMSD) values, over backbone atoms, for each monomer (indicated by colors in the legend above) of the indicated TRPA1 constructs.

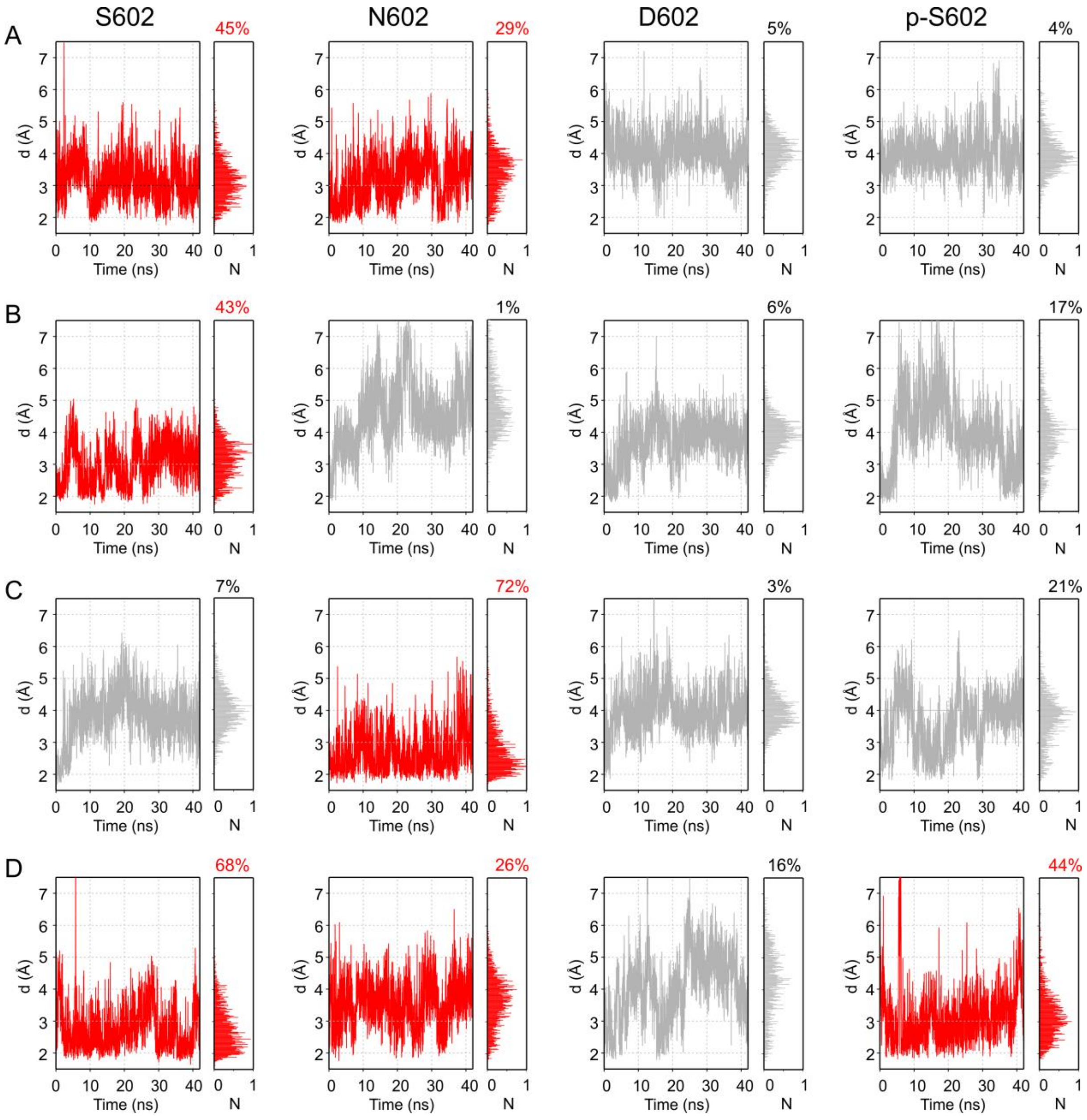

Figure S2. Molecular dynamics simulation results obtained for the TRPA1 models of wild-type (S602), N602, D602 and p-S602 based on the structure PDB ID: 6V9X. Time course of distances between Trp605 and Tyr662, measured as shown in Figure 4 from subunits A, B, C, D. Horizontal bar graphs are normalized all-point histograms produced from the distances shown in left panel over the simulation interval 15-42 ns. Percentages calculated from the histograms indicate the relative times (>25% in red) for which the distance between the two atoms is less than 3 Å.

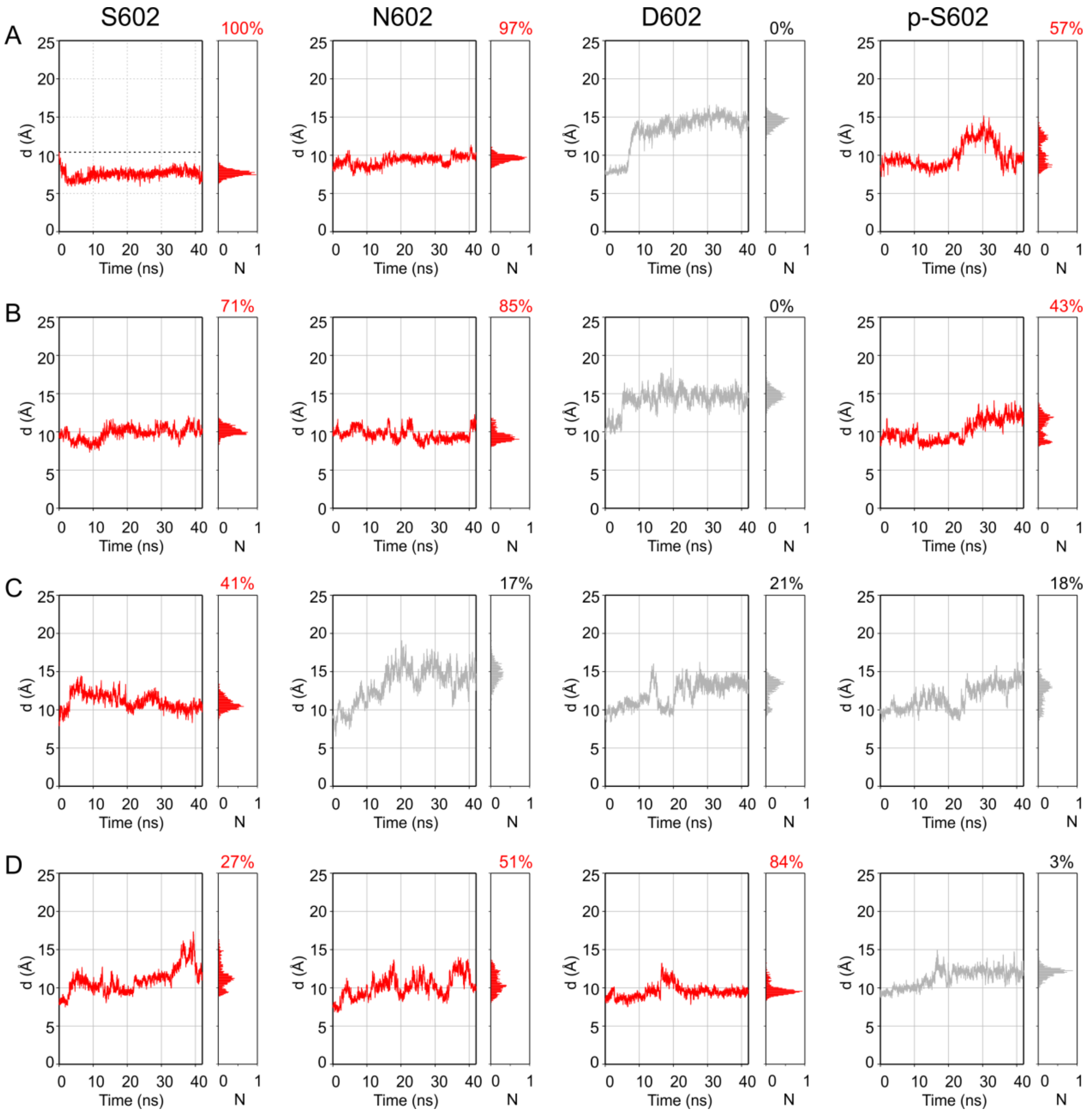

Figure S3. Molecular dynamics simulation results obtained for the TRPA1 models of the wild-type (S602), N602, D602 and p-S602 based on the structure PDB ID: 6V9X. Time course of distances between Lys671 and Glu987, measured between Cα atoms from subunits A, B, C, D. Horizontal bar graphs are normalized all-point histograms produced from the distances shown in left panel over the simulation interval 15-42 ns. The histograms of the wild-type subunits were fitted by Gaussian function and the threshold was set as the average ($\mu + \sigma$) = 10.4 Å. Percentages indicate the relative times (>25% in red) for which the distance between the two atoms is less than 10.4 Å.
